# Balanced pan-PPAR activator bezafibrate in combination with statin: comprehensive lipids control and diabetes prevention?

**DOI:** 10.1186/1475-2840-11-140

**Published:** 2012-11-14

**Authors:** Alexander Tenenbaum, Enrique Z Fisman

**Affiliations:** 1 Cardiac Rehabilitation Institute, Sheba Medical Center, 52621, Tel-Hashomer, Israel; 2 Sackler Faculty of Medicine, Tel-Aviv University, 69978, Tel-Aviv, Israel; 3 Cardiovascular Diabetology Research Foundation, 58484, Holon, Israel

**Keywords:** Atherogenic dyslipidemia, Bezafibrate, Combined fibrate/statin therapy, Metabolic syndrome, PPAR, Prevention, Residual cardiovascular risk, Type 2 diabetes

## Abstract

All fibrates are peroxisome proliferators-activated receptors (PPARs)-alpha agonists with ability to decrease triglyceride and increase high density lipoprotein- cholesterol (HDL-C). However, bezafibrate has a unique characteristic profile of action since it activates all three PPAR subtypes (alpha, gamma and delta) at comparable doses. Therefore, bezafibrate operates as a pan-agonist for all three PPAR isoforms. Selective PPAR gamma agonists (thiazolidinediones) are used to treat type 2 diabetes mellitus (T2DM). They improve insulin sensitivity by up-regulating adipogenesis, decreasing free fatty acid levels, and reversing insulin resistance. However, selective PPAR gamma agonists also cause water retention, weight gain, peripheral edema, and congestive heart failure. The expression of PPAR beta/ delta in essentially all cell types and tissues (ubiquitous presence) suggests its potential fundamental role in cellular biology. PPAR beta/ delta effects correlated with enhancement of fatty acid oxidation, energy consumption and adaptive thermogenesis. Together, these data implicate PPAR beta/delta in fuel combustion and suggest that pan-PPAR agonists that include a component of PPAR beta/delta activation might offset some of the weight gain issues seen with selective PPAR gamma agonists, as was demonstrated by bezafibrate studies. Suggestively, on the whole body level all PPARs acting as one orchestra and balanced pan-PPAR activation seems as an especially attractive pharmacological goal. Conceptually, combined PPAR gamma and alpha action can target simultaneously insulin resistance and atherogenic dyslipidemia, whereas PPAR beta/delta properties may prevent the development of overweight. Bezafibrate, as all fibrates, significantly reduced plasma triglycerides and increased HDL-C level (but considerably stronger than other major fibrates). Bezafibrate significantly decreased prevalence of small, dense low density lipoproteins particles, remnants, induced atherosclerotic plaque regression in thoracic and abdominal aorta and improved endothelial function. In addition, bezafibrate has important fibrinogen-related properties and anti-inflammatory effects. In clinical trials bezafibrate was highly effective for cardiovascular risk reduction in patients with metabolic syndrome and atherogenic dyslipidemia. The principal differences between bezafibrate and other fibrates are related to effects on glucose level and insulin resistance. Bezafibrate decreases blood glucose level, HbA1C, insulin resistance and reduces the incidence of T2DM compared to placebo or other fibrates. Currently statins are the cornerstone of the treatment and prevention of cardiovascular diseases related to atherosclerosis. However, despite the increasing use of statins as monotherapy for low density lipoprotein- cholesterol (LDL-C) reduction, a significant residual cardiovascular risk is still presented in patients with atherogenic dyslipidemia and insulin resistance, which is typical for T2DM and metabolic syndrome. Recently, concerns were raised regarding the development of diabetes in statin-treated patients. Combined bezafibrate/statin therapy is more effective in achieving a comprehensive lipid control and residual cardiovascular risk reduction. Based on the beneficial effects of pan-PPAR agonist bezafibrate on glucose metabolism and prevention of new-onset diabetes, one could expect a neutralization of the adverse pro-diabetic effect of statins using the strategy of a combined statin/fibrate therapy.

## Concept of the balanced pharmacological pan-PPAR activation

**Figure 1:**
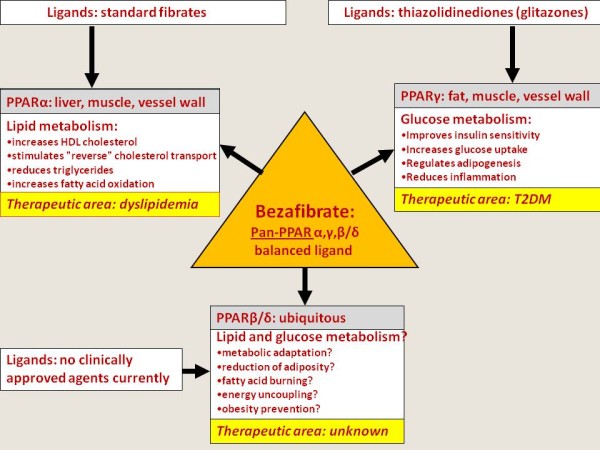
Bezafibrate activates all three PPAR subtypes (alpha, gamma and delta) at comparable doses and operates as a balanced pan-PPAR ligand.

All fibrates are peroxisome proliferators-activated receptors (PPARs)-alpha agonists with ability to decrease triglyceride and increase high density lipoprotein- cholesterol (HDL-C). However, bezafibrate, in comparison with other fibrates has a unique characteristic profile of action since it activates all three PPAR subtypes (alpha, gamma and delta) at comparable doses [1-5]. Therefore, bezafibrate operates as a pan-agonist for all three PPAR isoforms (Figure 1). Paradoxically, bezafibrate was serendipitously developed before the discovery of PPARs, but till now is the only clinically available pan-PPAR balanced ligand. What is the main distinctiveness of the PPARs from a clinical point of view?

PPAR alpha, activated by polyunsaturated fatty acids and fibrates, is implicated in the regulation of lipid metabolism, lipoprotein synthesis and metabolism, as well as inflammatory response in liver and other tissues. PPAR alpha plays a crucial role in regulating the β-oxidation of fatty acids, a major source of cellular energy. Consistent with this, PPAR alpha is highly expressed in tissues with high fatty acid oxidation (like liver, kidney and heart muscle), in which it controls a comprehensive set of genes that regulate most aspects of lipid catabolism. PPAR alpha activation increase HDL-C synthesis, stimulates "reverse" cholesterol transport and reduces triglycerides [6-8].

PPAR gamma regulates adipogenesis, lipid metabolism, glucose control, and inflammation/vascular pathways. Clinically, selective PPAR gamma agonists like thiazolidinediones (glitazones) are used to treat type 2 diabetes mellitus (T2DM). They improve insulin sensitivity by up-regulating adipogenesis, decreasing free fatty acid levels, and reversing insulin resistance. However, selective PPAR gamma agonists also cause water retention, weight gain, peripheral edema, and congestive heart failure. Such adverse effects may contribute to controversial cardiovascular outcomes despite apparent improvements in other risk factors [2,7,9,10].

Although PPAR beta/ delta agonists are not currently in clinical use (besides as a part of bezafibrate effects), their role in the whole body level seems very important. The expression of PPAR beta/delta in essentially all cell types and tissues (ubiquitous presence) suggests its potential fundamental role in cellular biology and possible widespread effects of its agonists [11,12]. Particularly, selective overexpression of a constitutively active form of PPAR beta/delta in mouse adipose tissue induces significant weight loss and protects against the obesity and dyslipidemia induced by a high-fat diet [13]. This PPAR beta/ delta effect correlated with enhancement of fatty acid oxidation, energy consumption and adaptive thermogenesis. This change may explain why mice that overexpress PPAR beta/ delta in skeletal muscle can run twice the distance of control mice [14]. Together, these data implicate these PPAR in fuel combustion and suggest that pan-PPAR agonists that include a component of PPAR beta/delta activation might offset some of the weight gain issues seen with selective PPAR gamma agonists [15], as was demonstrated in bezafibrate studies [16].

The PPARs alpha and gamma are therapeutic targets for hypertriglyceridemia and insulin resistance, respectively. Evidence is now emerging that the PPARbeta/delta isotype is a potential pharmacological target for the treatment of disorders associated with metabolic syndrome. PPAR beta/delta activation increases lipid catabolism, improves the serum lipid profile and insulin sensitivity in several animal models. In addition, PPAR beta/delta ligands prevent weight gain and suppress macrophage-derived inflammation. These data are promising and indicate that PPAR beta/delta ligands may become a therapeutic option for the treatment of metabolic syndrome [17,18].

Suggestively, on the whole body level all PPARs acting as one orchestra and balanced pan-PPAR activation seems as an especially attractive pharmacological goal. Conceptually, we were the first who proposed an idea of pan-PPAR activation: combined PPAR gamma and alpha action can target simultaneously insulin resistance and atherogenic dyslipidemia [2,6], whereas PPAR beta/delta properties may prevent the development of overweight which typically accompanies selective PPAR gamma ligands.

## Balanced pan-PPAR activator bezafibrate: effects in patients with atherogenic dyslipidemia and metabolic syndrome

Hence, what data have we while looking on pan-PPAR balanced activator bezafibrate? As all fibrates (PPAR alpha activators), it significantly reduced plasma triglycerides and increased HDL-C level (considerably stronger than other major fibrates) [19-21]. Bezafibrate significantly decreased the prevalence of small, dense LDL particles, remnants [22] and induced atherosclerotic plaque regression in thoracic and abdominal aortas [23]. In addition, bezafibrate has important fibrinogen-related properties and anti-inflammatory effects. It is well established that fibrinogen is an independent predictor of cardiovascular events in patients with coronary artery disease [24]. Bezafibrate treatment lowers fibrinogen concentrations and reduces other markers of systemic inflammation (like tumor necrosis factor (TNF)-alpha and interleukin (IL)-6 and C-reactive protein) [25-27]. As a result, endothelial function was improved by bezafibrate [28-30] but probably in a less extend than by high dose atorvastatin [31].

In clinical trials bezafibrate, as all fibrates, was highly effective in patients with atherogenic dyslipidemia (presented by low HDL-C and increased triglycerides). In patients without dyslipidemia this favorable effect was absent and the same event rate was observed in both bezafibrate and placebo groups. Consequently, the main determinant for the overall results of the trial was mainly dependent on the number of the appropriately included patients [19]. For example, in the Bezafibrate Infarction Prvention (BIP) study about 50% of patients did not need fibrates at all. In addition, significantly more patients from the placebo group were “under covered” treated with statins (it was a violation of the study protocol). As a result, after a mean 6.2 year follow-up bezafibrate effect did not reach significance in the overall population (9.4% risk reduction, p=0.24). However, in appropriate patients with atherogenic dyslipidemia bezafibrate lead to 42% risk reduction (p=0.02) [19,20]. Furthermore, after a mean 8.2 year follow-up bezafibrate benefit was significant even in the overall population (17% risk reduction, p=0.03) [32].

There are few direct “head to head” statin vs. bezafibrate comparisons in terms of cardiovascular outcomes. Intermediate-size (274 patients) randomized controlled trials have demonstrated that bezafibrate was significantly better than pravastatin (a relatively weak statin) in reduction of cardiovascular events [33].

Because pan-PPAR modulation is a key event in the development of the metabolic syndrome [34], it was of special interest to evaluate bezafibrate effects in this syndrome. In patients with metabolic syndrome, bezafibrate treatment was associated with a significant (29%) reduced risk of any myocardial infarction (MI) and 33% reduced risk of non-fatal MI. The early decrease in MI incidence was reflected later in a tendency to reduced cardiac mortality. Of note, among patients with augmented features of metabolic syndrome (4–5 risk factors for metabolic syndrome) a marked 56% reduction in cardiac mortality on bezafibrate was observed [16].

**Figure 2:**
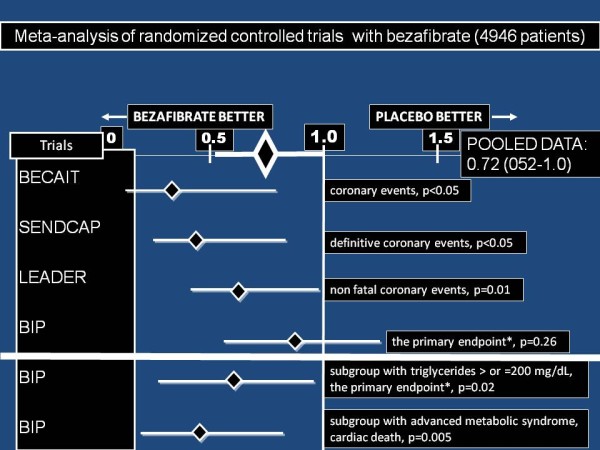
**Meta-analysis of all available 4 randomized control trials with bezafibrate[**[20,21,35,36]**] in “overall populations” (4946 patients), based in part on the comprehensive meta-analysis of Jun and colleagues [**[37]**]: 28% coronary events risk reduction was observed (relative risk 0.722, confidence interval 0.518-1.005).** This effect was significantly augmented (shown below the bold white line) in the subgroups of appropriate patients with metabolic syndrome and dyslipidemia [19,20].

Figure 2 illustrates meta-analysis of all available 4 randomized control trials with bezafibrate (about 5000 patients) in “overall populations” [20,21,35,36], based in part on the comprehensive meta-analysis of Jun and colleagues [37]: 28% coronary events risk reduction was observed. As mentioned above, this effect was significantly augmented in appropriate patients with metabolic syndrome and dyslipidemia [19,20].

## Balanced pan-PPAR activator bezafibrate: effects on glucose metabolism and prevention of T2DM

The principal differences between bezafibrate and other fibrates are related to its effects on glucose level and insulin resistance. A large number of studies showed beneficial effects of bezafibrate on glucose and insulin metabolism [25,38-46]. These effects could be primarily related to the direct influence of bezafibrate on insulin sensitivity via PPAR gamma. However, additional PPAR alpha – mediated effects on lipids metabolism may be also important.

Hyperlipidemia, free fatty acids (FFAs) overload and lipid accumulation in non-adipose tissues influence both insulin action and insulin secretion and are frequently associated with insulin resistance and the development of T2DM [47-52]. We have previously shown that insulin resistance is significantly correlated with triglycerides level. Triglyceride-lowering and HDL-C–raising effects of bezafibrate lead to decreased systemic availability of fatty acids, diminished fatty acid uptake by muscle with improvement of insulin sensitization, contributing also to a reduction of plasma glucose levels [40,42]. However the role of FFAs extends beyond their ability to induce or exacerbate insulin resistance and inflammation: they may contribute directly to the deterioration of beta cell function that accompanies the development of diabetes [53-56]. Lipotoxicity and glucotoxicity share many common features, and FFAs and glucose metabolism are intimately linked through their ability to act as competing oxidative substrates [48]. Probably, reducing the chronic secretory demands for pancreatic beta-cell by improving insulin sensitivity and direct reducing of triglycerides and FFA accumulation in pancreatic islets can explain preservation of beta cell function in patients with T2DM treated with bezafibrate.

Bezafibrate has a modest but significant beneficial effect on HbA1C [41,43]. For example, in the recent large Japanese the J-BENEFIT study HbA1c decreased significantly by about −0.8% [43]. This effect was observed irrespective of diabetes drug administration, but with a strong positive relationship between triglyceride and HbA1c reduction. In the BIP, bezafibrate reduces the incidence and delays the onset of T2DM vs. placebo among different high-risk populations: patients with impaired fasting glucose (30% reduction) [44] and obese patients even with normal fasting glucose levels (42% reduction) [45]. A strong independent support for BIP was found in a large British study of 16352 fibrate users during 5-year follow-up: 12161 of patients used bezafibrate and 4191 of patients used other fibrates [46]. Bezafibrate was associated with reduced hazard for diabetes of 41%; hazard for progression to use of antidiabetic medication was by 46% less and progression to insulin by 22% less in patients with bezafibrate.

In contrast to patients treated with selective PPAR gamma activators, there was no significant change in mean body mass index (BMI) values in the bezafibrate treated patients during the follow-up [19]. Probably, prevention of weight gain could be explained by the PPAR beta/delta mediated effects of bezafibrate. In addition, bezafibrate significantly increased adiponectin levels both in humans and rodents. This effect was mediated mostly via PPAR alpha, but also partially via PPAR gamma [57]. Therefore, a pharmacological intervention that simultaneosly influences lipids and glucose metabolism can be particularly effective to reduce the incidence and delay the onset of T2DM in appropriate high risk patients. These data support our suggestion that on the whole body level a balanced synergism of all PPARs could be justified.

## Bezafibrate in combination with statin: current concepts, evidences and suggestions

Currently statins are the cornerstone of the treatment and prevention of cardiovascular diseases related to atherosclerosis [58-60]. However, despite the increasing use of statins as monotherapy for LDL-C reduction, a significant residual cardiovascular risk is still present in patients with atherogenic dyslipidemia and insulin resistance which is typical for T2DM and metabolic syndrome [61-71]. The risk associated with high triglycerides and low HDL may be eliminated by bezafibrate. Among major fibrates, bezafibrate appears to have the strongest [42,47] effect on HDL-C.

Intensive statin therapy increases the possibility of side effects (elevations of liver enzymes, muscle aches and cognitive decline). Recently, concerns raised regarding the development of diabetes in statin-treated patients. Multiple studies showed that most popular statins - simvastatin, atorvastatin, and rosuvastatin - have deleterious effects on glycemic control [72-83]. For example, in the CORONA trial [77], statin users were at a 1.13-fold (95% CI, 0.86–1.50) greater risk of developing new-onset diabetes than patients taking placebo. In the JUPITER trial [78], the relative risk was even higher (1.25 [95% CI, 1.05–1.49]). In the SPARCL (Stroke Prevention by Aggressive Reduction in Cholesterol Levels) trial [81], new-onset T2DM developed in 166 of 1,905 patients randomized to atorvastatin 80 mg/day and in 115 of 1,898 patients in the placebo group (8.71% vs. 6.06%, adjusted HR: 1.37, 95% CI: 1.08 to 1.75, p = 0.011). It should be pointed out that a newest statin – pitavastatin – seems to be better in this issue in the post-hoc LIVES study sub-analysis [84]. Still, the cardiovascular benefits of statin therapy clearly outweigh the risk of developing diabetes [60,82]. However, the data suggest the need to make patients aware of this possible risk and to monitor patients for eventual development of diabetes, especially on intensive-dose therapy. In addition, concerns regarding new-onset diabetes should not be neglected due to the serious economic burden which this condition presents now.

Of course, the importance of the intensive lifestyle changes (diet, weight reduction and physical activity) should be emphasized irrespective from the pharmacological interventions. However, as compared with statin monotherapy (effective mainly on LDL-C levels and plaque stabilization), the association of a statin with bezafibrate will also have a major impact on triglycerides, HDL and LDL particle size [20,22,58,85]. Moreover, based on the beneficial effects of pan-PPAR agonist bezafibrate on glucose metabolism and prevention of new-onset diabetes (about 30-40% risk reduction), one could expect neutralizing of the adverse pro-diabetic effect of statins (up to 25% increased risk). Therefore, a combined bezafibrate/statin therapy will be more effective in achieving a comprehensive lipid control, residual cardiovascular risk reduction and theoretically could prevent statin induced new-onset diabetes.

Though muscle pain and myositis is an issue in statin/fibrate treatment, adverse interaction appears to occur to a significantly greater extent when gemfibrozil is administered. Bezafibrate seems to be safer and better tolerated [86-96]. Particularly, plasma concentration of statins are markedly increased by gemfibrozil but not by bezafibrate [92]. However, safety concerns about gemfibrozil may lead to unjustified precautions regarding bezafibrate administration and therefore diminish the use of this useful agent.

The Multicentre double-blind The Fluvastatin Alone and in Combination Treatment (FACT) study examined the effects on plasma lipids and safety of a combination of fluvastatin and bezafibrate during 24 weeks in 333 patients with coronary artery disease and mixed hyperlipidaemia [95]. Bezafibrate alone and fluvastatin+bezafibrate combinations resulted in greater increases in HDL-C and decreases in triglycerides compared with fluvastatin alone (P<0.001). Fluvastatin (40 mg)+bezafibrate was the most effective for all lipid parameters with a decrease from baseline at endpoint in LDL-C of 24%, a decrease in triglycerides of 38% and an increase in HDL-C of 22%. All treatments were well tolerated with no increase in adverse events for bezafibrate-based combination therapy versus monotherapy. No clinically relevant liver (aspartate aminotransferase [ASAT] or alanine aminotransferase [ALAT]) greater than three times the upper limit of normal) or muscular (creatine phosphokinase (CPK) greater than four times the upper limit of normal) laboratory abnormalities were reported.

The pooling data from 10 studies that included 1,018 patients treated with a combination of fluvastatin with fibrate (bezafibrate 493 patients, fenofibrate 158 patients, or gemfibrozil – 367 patients), have suggested that bezafibrate is the relatively safer fibrate for combination treatment [96]. For these patients, the mean (very high) dose of fluvastatin was 56 mg. The mean exposure was 38 weeks. Two patients (0.2%) treated with the combined fluvastatin–fibrate therapy had a creatine kinase level 10 times the upper limit of normal. One patient was on a combination of fluvastatin 80 mg + gemfibrozil 1,200 mg, and the other patient was on fluvastatin 20 mg + fenofibrate 200 mg. Bezafibrate, which was used 3 times more than fenofibrate, was harmless in this analysis.

The safety and efficacy of combined bezafibrate-simvastatin therapy was evaluated in 49 patients with diet-resistant mixed hyperlipidaemia [93]. After a two-month placebo phase, patients were randomized to receive either Bezafibrate Slow Release (SR) 400 mg or simvastatin 20 mg followed by three months of combination therapy. Compared with simvastatin monotherapy, the combined therapy induced further reductions in triglycerides (by 26%, p = 0.0003) and apoB (by 11 mg/dl, p = 0.03) and an increase in apoA1 (by 21 mg/dl, p = 0.0008). Symptomatic and biochemical adverse events did not occur more frequently on combined drug therapy than on monotherapy. The combination of bezafibrate and simvastatin was more effective in controlling mixed hyperlipidaemia than either drug alone and did not provoke more adverse events. Despite of the strong theoretical background, there are few hard outcome data regarding combined bezafibrate and statin treatment: only one small randomized, controlled clinical trial and a number of the observational studies. Gavish et al. [94] studied the efficacy and safety of bezafibrate and simvastatin combination therapy for diabetic dyslipidaemia. All patients (148) were on single medication for a period of 6 months (100 on simvastatin 20 mg day and 48 on bezafibrate SR 400 mg day), followed by a period of 12 months on combined therapy for all. The simvastatin and bezafibrate combination was found to be more efficacious than a single medication for treatment of diabetic dyslipidemia, as evidenced by improvement in the lipoprotein profile, reductions in Lp(a) and fibrinogen, and almost no clinically significant side-effects. Side-effects included two patients who developed myopathy when on the combined regimen and one on the single statin regimen. Plasma creatinine phosphokinase (CPK) levels increased (but remained within the normal range) in most of the patients on combination therapy. Most of the cardiovascular events occurred within the first 6 months of the study, whilst the patients were on a single drug (12 events, 8.1%). There was a significant reduction in the event rate during the year whilst the patients were on the combination therapy (only two events – 1.4 %).

Recently, new data regarding statin/fibrate combination were published using the high quality comprehensive nationwide Acute Coronary Syndrome Israeli Surveys (ACSIS) registry [97]. There were 8545 patients treated with statin alone and 437 patients treated with a statin/fibrate combination (mainly bezafibrate). Development of 30-day major acute coronary event (MACE) - primary end-point - was recorded in 6.0% patients from the statin monotherapy group vs. 3.2% from the combination group, (p=0.01). The 30-day re-hospitalization rate was also significantly lower in the combination group. Kaplan-Meier analysis of total mortality during one year was close to significance in favor of the combination (p=0.066). Multivariate analysis identified the fibrate/statin combination as an independent predictor of 46% reduced risk of MACE in overall population (p=0.03).

Madrid-Miller et al. in a small randomized controlled trial [26] investigated the impact of the addition of bezafibrate to statin treatment in 50 patients with hyperfibrinogenemia and ST-elevation acute MI. Patients were randomized into two groups (25 patients in each): bezafibrate 400 mg (group I) and conventional therapy (group II). All patients were treated with statins. Primary endpoint was decrease of fibrinogen concentrations. Secondary endpoints were recurrence of angina or infarction, left ventricular failure and combined end points during hospitalization. Fibrinogen concentrations were lower at hospital discharge in group I than in group II (532.42 ± 129.6 vs. 889 ± 127.32 mg/dl in group II, p <0.0001). Secondary endpoints were more frequent in group II than in group I: angina (56% vs. 4%, RR 0.071 [0.010-0.503], p <0.0001), left ventricular failure (24% vs. 4%, RR 0.167 [0.022-1.286], p = 0.049) and combined endpoints (76% vs. 8%, RR 0.105 [0.027-0.405], p <0.001). Therefore, bezafibrate/statin treatment was a safe and was associated with a lower incidence of MACE compared with statins alone. Although in clinical trials the rate of adverse events on combination was not significantly greater compared with monotherapy, clinical and laboratory monitoring of patients who receive combined treatment should be recommended.

## Conclusions

The balanced pan-PPAR activator bezafibrate simultaneously targets atherogenic dyslipidemia and insulin resistance without development of overweight; it reduces plasma triglycerides and glucose levels, increases HDL-C and decreases the prevalence of small, dense LDL particles. As a result of its concomitant effects on lipid and glucose metabolism, bezafibrate reduces the incidence and delays the onset of T2DM in high risk patients. In patients with atherogenic dyslipidemia and/or metabolic syndrome bezafibrate is consistently associated with a reduced risk of cardiovascular events. Combined bezafibrate/statin therapy is more effective in achieving a comprehensive lipid control and residual cardiovascular risk reduction. Based on the beneficial effects of this pan-PPAR agonist on glucose metabolism and prevention of new-onset diabetes, a neutralization of the adverse pro-diabetic effects of statins by using a combined therapy could be expected.

## Abbreviations

ACSIS: Acute Coronary Syndrome Israeli Surveys; BIP: Bezafibrate Infarction Prvention study; BMI: body mass index; FFAs: free fatty acids; HDL-C: high density lipoprotein- cholesterol; LDL-C: low density lipoprotein- cholesterol; MACE: major acute coronary event; MI: myocardial infarction; PPAR: peroxisome proliferator-activated receptor; T2DM: type 2 diabetes mellitus.

## Competing interests

AT received speaker fee and travel expenses support from Abbott, Tribute, Novartis and Merck.

EZF declares that he has no competing interests.

## Authors’ contribution

Both authors have equally contributed in the conception and drafting of the manuscript. Both authors read and approved the final manuscript.
